# A new genus and species of Coenosiini from Costa Rica (Diptera, Muscidae, Coenosiinae)

**DOI:** 10.3897/zookeys.321.5443

**Published:** 2013-08-05

**Authors:** Márcia Souto Couri, Claudio José Barros de Carvalho

**Affiliations:** 1 Department of Entomology, Museu Nacional, Quinta da Boa Vista, São Cristóvão, Rio de Janeiro, 20.940–040, RJ, Brazil; 2 Department of Zoology, Universidade Federal do Paraná, 81531-980, PO Box 19020, Curitiba, PR, Brazil; 3 Research fellow, CNPq, Brazil

**Keywords:** Morphology, Neotropical region, *Palpilongus* gen. n., *Palpilongus bifurcus* sp. n., taxonomy

## Abstract

*Palpilongus* **gen. n**. is herein described for one species – *Palpilongus bifurcus* **sp. n**., from Costa Rica, based on male and females. The striking morphological characters of the species – palpus very long, about as long as prementum; upper calypter truncate and very short and setae of male sternite 5 bifurcated, confirm that this new species is also a new genus in the tribe Coenosiini. Male and female terminalia were dissected and illustrated.

## Introduction

The Coenosiinae, a subfamily of the Muscidae, comprises the tribes Limnophorini and Coenosiini, both with genera in the Neotropical region (Couri and de Carvalho 2002). Members of the Coenosiini tribe share: (i) eyes usually dichoptic in both sexes, (ii) lower proepimeral seta downward directed, (iii) katepisternal setae 1:1:1, forming an equilateral triangle, (iv) hind tibia with at least one anterodorsal seta and (v) male hypandrium tubular and elongated (Couri and Carvalho 2002). The tribe is monophyletic, and the arrangment of the katepisternal setae was the single synapomorphy that supported the monophyly of the Coenosiini in Couri and Pont (2000) analysis. A downcurved lower proepimeral setae was first pointed out by Malloch (1934) as another synapomorphy for Coenosiini, but this character also appears in *Agenamyia* Albququerque temporarely transferred by Couri and Pont (2000) to the Limnophorini tribe, of the Coenosiinae subfamily.

Most of the species are known as predators of other insects and some play an important role as potential biocontrol agents, as, for instance, *Coenosia attenuata* Stein (Couri and Salas 2010).

Coenosiini is the largest tribe of Coenosiinae, with 29 genera in the world, 15 in the Neotropical region. Four are currently known from Costa Rica: *Bithoracochaeta* Stein, 1911, *Cordiluroides* Albuquerque, 1954, *Neodexiopsis* Malloch, 1920 and *Schoenomyza* Haliday, 1833 (de Carvalho et al. 2005; Couri et al. 2006).

The aim of the present contribution is to describe a new Coenosiinae species from Costa Rica, and to ascribe it to a new genus based on unique combination of characters.

## Materials and methods

This study was based on one male and eight females specimens from Costa Rica in the collection of the Instituto Nacional de Biodiversidad (INBIOInstituto Nacional de Biodiversidad, Costa Rica). Two female paratypes (one each) will be deposited at the Museu Nacional, Universidade Federal do Rio de Janeiro (MNRJMuseu Nacional, Universidade Federal do Rio de Janeiro) and at the Department of Zoology of the Universidade Federal do Paraná (DZUPDepartment of Zoology of the Universidade Federal do Paraná). Holotype and the remaining paratypes remain at INBIO. Terminology follows McAlpine (1981) except for postpedicel instead of antennal flagellomere, as we followed Stuckenberg (1999).

The terminalia were macerated in a 10% potassium hydroxide solution at room temperature for 24 hours. They were then dissected in glycerol and stored in a microtube with the specimen. Color photos were taken with Syncroscopy, JVC Auto-Montage with a Leica MZ 16 optical microscope.

## Taxonomy

### Genus Palpilongus gen. n.

http://zoobank.org/40B2A451-4FEA-408A-8509-0FBE48BA61AF

http://species-id.net/wiki/Palpilongus

#### Type-species.

*Palpilongus bifurcus* sp. n., by present designation

#### Diagnosis.

**Figure 1.**
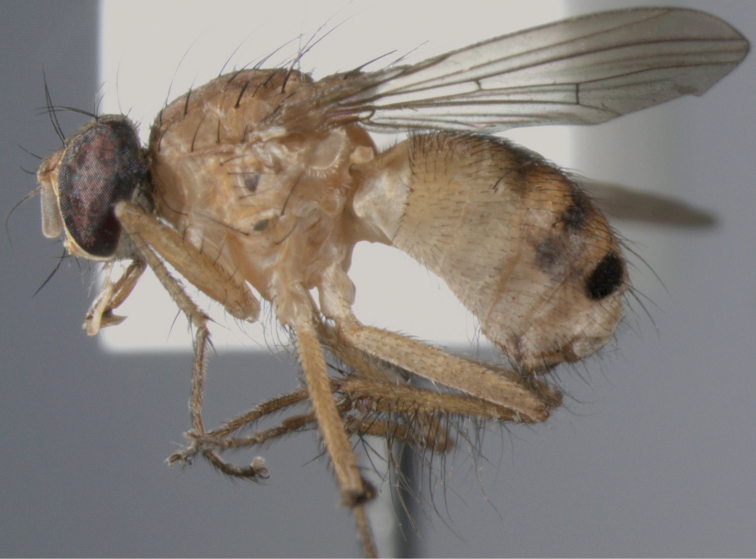
*Palpilongus bifurcus* gen. n. et sp. n. (holotype), habitus in lateral view.

**Figure 2.**
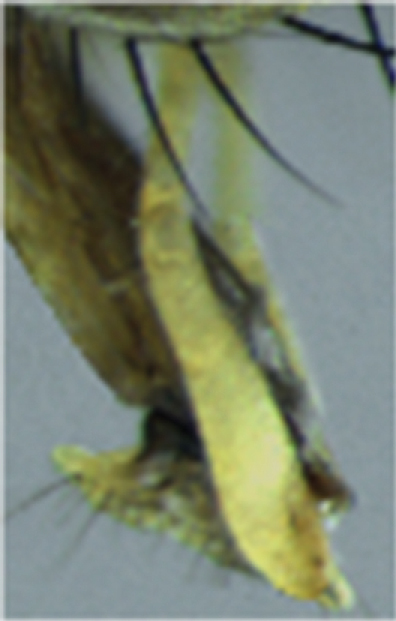
*Palpilongus bifurcus* gen. n. et sp. n. (holotype), mouthparts and palpus in lateral view.

(Fig. 1) Male dichoptic; eye bare, separated by about 1/3 of head width in both sexes; palpus flattened, very long with length equal to that of the prementum (Fig. 2); proepimeral seta oriented downwards; notum and pleurae with very few setulae; presutural acrostichal setae developed; dorsocentral setae 1 + 3; katepisternals 1 + 1 + 1 forming an equilateral triangle; upper calypter truncate and very short; wing veins bare; male hind tibia with many rows of fine and long anterodorsal, dorsal and posterodorsal setae; sternite 1 bare; setae on sternite 5 bifurcated; hypandrium tubular in male; ovipositor long in female.

#### Etymology.

Derived from the Latin words *palpus* and *longus*, the genus name refers to the long palpus.

#### Discussion.

In the current classification of Muscidae, the new genus falls in the tribe Coenosiini of the Coenosiinae. In both keys to muscid genera identification of de Carvalho and Couri (2002) for the Neotropical region and Savage and Vockeroth (2010) for Central America, the new genus approaches *Neodexiopsis* and can be separated by the following couplet:

**Table:** 

1	Palpus short, much shorter than prementum length; upper calypter glossiform and not very short; setae on sternite 5 not bifurcated	*Neodexiopsis* Malloch
–	Palpus very long about as long as prementum; upper calypter truncate and very short; setae on sternite 5 bifurcated	*Palpilongus* gen. n.

The new genus was added to the cladistic analysis of the world Coenosiini (Couri and Pont 2000). The analysis positioned *Palpilongus* in the same clade of *Cordiluroides* + *Neodexiopsis* + *Haroldopsis* Albuquerque (synonymyzed with *Neodexiopsis* in the referred cladistic analysis), based on one synapomorphy - presence of three preapical setae on mid femur. The new genus was supported by the following synapomorphies: hairs on arista at most equal to basal width of arista; fronto-orbital plate with no setulae; colour of thorax and abdomen shinning undusted; notum almost bare, with very few ground setulae; lateral seta on scutellum present and hind tibia with a preapical posterodorsal seta at apical fourth.

*Neodexiopsis* and *Cordiluroides* are differently represented in the Neotropical region. *Neodexiopsis* is a speciose muscid genus, with about hundred described species found throughout the region, while *Cordiluroides* have a more restricted geographic distribution (Mexico, Costa Rica and Brazil) and is known by 6 species.

Most of the *Neodexiopsis* species have light brown to dark brown bodies, grey pollinose, and yellow to brown legs. Adults are small to medium sized predatory flies that inhabit forests or open habitats. Similarly to other genera of the Coenosiini tribe, the characteristic chaetotaxy of the hind tibia can distinguish one from each other. *Neodexiopsis* species can be recognized by the presence of one anterodorsal, one posterodorsal and one anteroventral setae in hind tibia, all near middle, the last one shorter and can be absent in some species. Besides this, the palpus is short and filiform, the upper calypter is glossiform and not reduced and the setae on sternite 5 are not bifurcated, as in *Palpilongus* gen. n.

*Cordiluroides* species can be recognized by the very high insertion of the antenna (very above the mid level of the eye), palpus short and slender, presence of only one pair of postsutural intra-alar seta, upper calypter transverse, hind tibia with one median anterodorsal, one posterior submedian and one posterodorsal supramedian setae and setae on sternite 5 not bifurcated. The genus was recently recorded from Costa Rica, on the base of three species (Couri et al. 2006).

### Palpilongus bifurcus sp. n.

http://zoobank.org/C1D0584D-A01B-4539-8A97-B60DCF53A209

http://species-id.net/wiki/Palpilongus_bifurcus

Figs 1 –9

#### Type material.

Holotype male: COSTA RICA: Prov. Guana [Guanacaste], Estation Pitilla 9 Km. S. de Santa Cecilia, 700m, DIC 1994, P. Rios, LN 329950 380450 #4372 [INBIO code collection number] (deposited at INBIO). Paratypes: Same locality as holotype, ix.1994, LN 330200_380200 #3206, 1 female (INBIO), #3294, 1 female (INBIO); Prov. Alajuela, Upala, Bijagua, Alb. Heliconias, 700m, 11–26.i.2000, J. D. Gutiérrez, Agua miel, L_N_299800_43800 #56263, 1 female (MNRJ), #56263, 1 female (INBIO); Prov. Guanacaste, Rio San Lorenzo, Tierras Morenas, 1050m, x.1995, G. Rodriguez, L_N_287800_427600 #6405, 1 female (DZUP), # 6405, 1 female (INBIO); Prov. Punta [Puntarenas], Est La Casona, R.B. Monteverde, A.C. Arenal, 1520m, i.1994, N.G. Obando, LN 253250_449700 #2606, 1 female (INBIO); N. P. Heredia Prov., Transecto, Braulio Carrillo, x.1989, 1500, R. Aguillar & M. Zumbado, 1 female (INBIO).

#### Description.

**Male**. Body length – 3.8 mm. Wing length – 4.0 mm.

*Head*. Dichoptic. Ground-color yellow. Eye bare. Fronto-orbital plate, parafacial, face and gena golden pruinose. Frons brown-reddish about 1/3 of head width. Three pairs of frontal setae intercalated with shorter setae, the upper frontal setae oriented backwards. Ocellar setae strong. Antenna with pedicel yellow and postpedicel brownish, about 3.8 times the length of the pedicel. Arista with very short setulae. Gena thin. Palpus yellow, very long, as long as proboscis, enlarged toward apex. Labellum not reduced, developed and without teeth.

**Figure 3.**
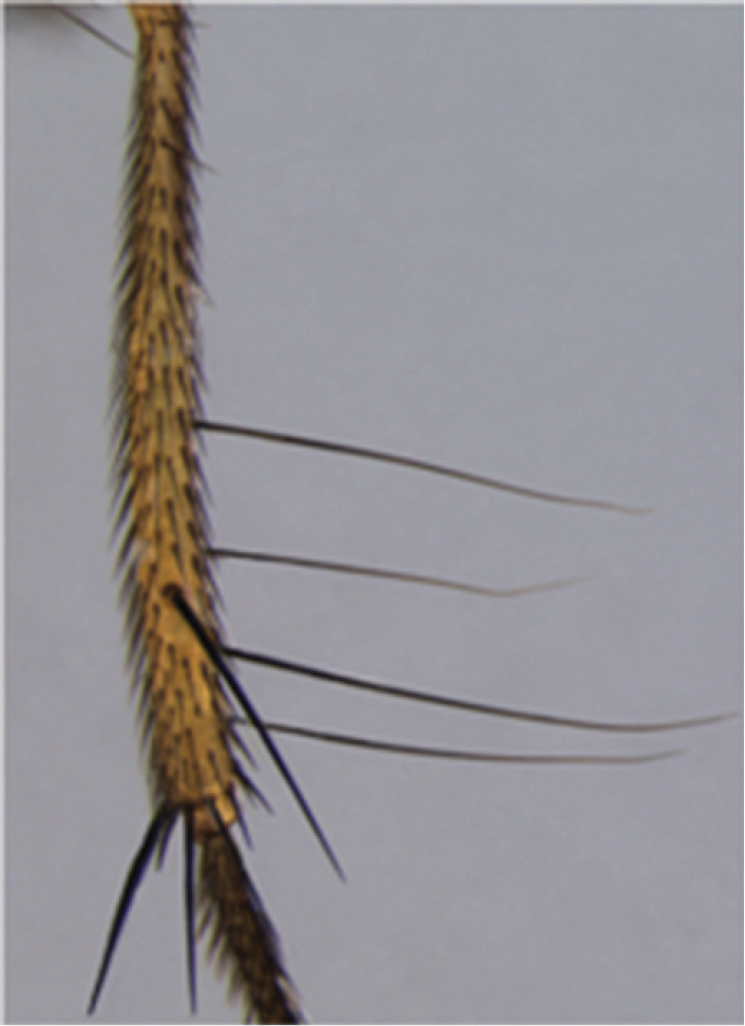
*Palpilongus bifurcus* gen. n. et sp. n. (holotype), mid tibia.

*Thorax*. Color yellow, with no marks. Acrostichal setae in front of suture in 3 irregular rows and after the suture in 1-2 rows, with some scattered cilia close to scutellar suture; notum with very few scattered cilia that increase somewhat in number on the scutellum; dorsocentrals 1+3, all long; intra-alars 1+2, supra-alars 2; postpronotals 2; prealar absent. Notopleuron without covering cilia and with two setae similar in size. Anepisternum with one row of six setae, one cilium on upper anterior margin and few scattered cilia on upper half. Prosternum bare, proepisternal depression bare, proepisternal setae oriented downwards. Katepisternals 1+1+1 and with scattered setulae. Meron and katepimeron bare. Scutellum with 1 short sub-basal seta, one lateral long and one apical a little shorter than the lateral one. Legs yellow. Fore femur with rows of posterodorsal and posteroventral setae. Fore tibia with 1 long median posterior seta, one strong dorsal seta on apical fourth, a pre-apical posterodorsal seta and an apical posteroventral seta. Tarsus with a long apical seta at base. Mid femur with short and thin row of scattered setae, ending with a longer seta; two pre-apical posterior setae. Mid tibia with 4 long dorsal setae on apical half (Fig. 3), one long and strong anterodorsal seta on apical third and long apical setae on each, the anterior, anteroventral and ventral, the last of which the longest and strongest. Hind femur with 2–3 fine anterodorsal and posterodorsal setae on apical third and with 3 preapical setae (anterodorsal, dorsal and posterodorsal). Hind tibia with many series of fine and long anterodorsal, dorsal and posterodorsal setae and with a long and strong apical ventral seta. Wing slightly infuscated. Vein M straight. Calypters yellow. Knob of halter yellow.

**Figure 4.**
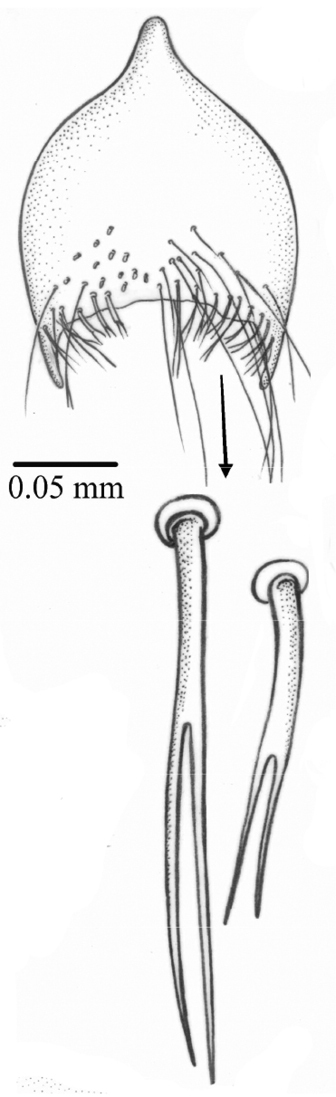
*Palpilongus bifurcus* gen. n. et sp. n. (holotype), sternite 5 with bifurcate setae in detail.

*Abdomen*. Elongate-cylindrical. Ground-color yellow with black round lateral marks on tergites 3–5. Sternite 1 bare. Sternite 5 with setae on apical third, the marginal ones bifurcated (Fig. 4).

**Figures 5–6.**
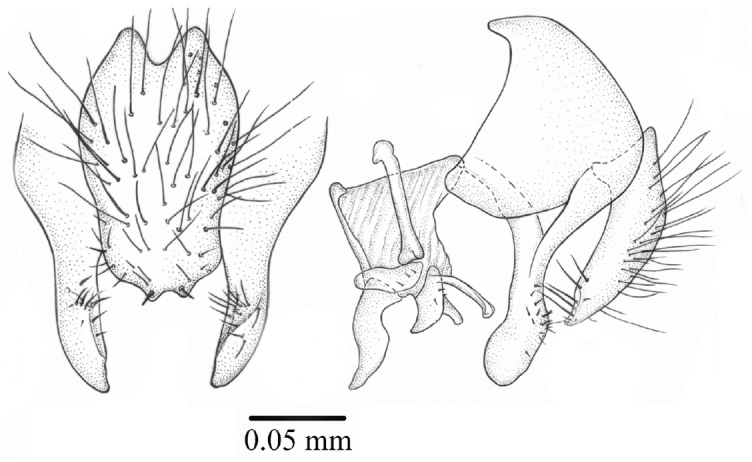
*Palpilongus bifurcus* gen. n. et sp. n. (holotype), cercal plate and surstyli **5** dorsal view **6** lateral view.

*Terminalia*. Cercal plate longer than wider, with setae on all surfaces and two small lateral spines on posterior margin (Figs 5–6); hypandrium tubular and phallic complex structures as in Fig. 5.

**Figure 7.**
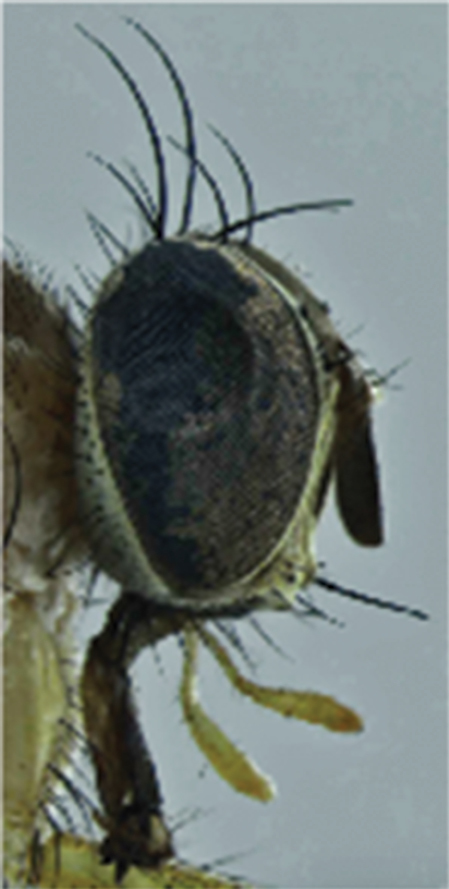
*Palpilongus bifurcus* gen. n. et sp. n. (female paratype), head in lateral view.

**Female**. Length of body: 4.5–5.3 mm. Length of wing, 4.8–5.5 mm. Differs from male as follows: proboscis with reduced labellum and strong teeth (Fig. 7). Mid femur has a median anterior seta. Mid tibia with one seta anterior to anterodorsal sub-basal seta and one supramedian posterior seta; hind femur with an anterodorsal and an anteroventral row of scattered setae and two long and thin ventral setae on the middle third. Hind tibia without the long setae as in male and with two anterodorsal setae at the limit of the median 1/3 and one submedian posterodorsal seta.

#### Ovipositor.

**Figures 8–9.**
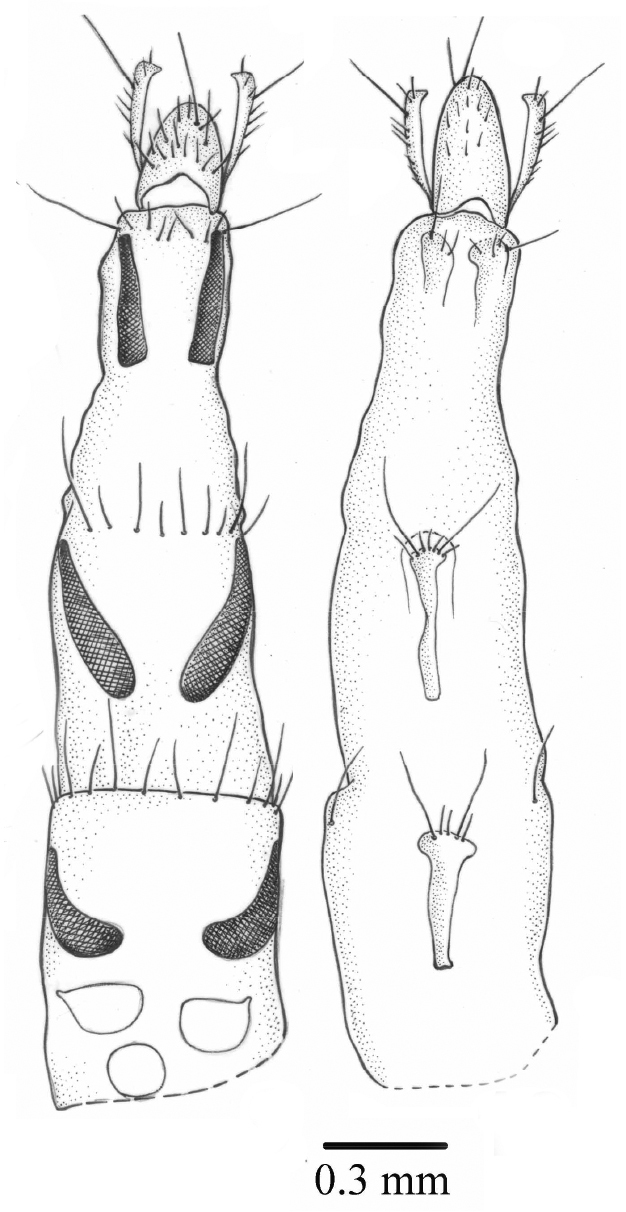
*Palpilongus bifurcus* gen. n. et sp. n. (female paratype), ovipositor **8** dorsal view and spermathecae **9** ventral view.

Long, tergites 6–8 long and thin, covered with microtrichia, sternites 6–8 undivided, hypoproct long, cerci long (Figs 8–9). Three round spermathecae.

#### Distribution.

Known only from Costa Rica.

#### Remarks.

The male and the female have some marked differences, mostly in mid and hind leg chaetotaxy, in which only the male has mid tibia with four long dorsal setae on apical half and hind tibia with many series of fine and long anterodorsal, dorsal and posterodorsal setae. The different shape of the proboscis suggests that feeding habits differs between males and females: the female certainly is predator as most species of Coenosiini with reduced labellum and developed teeth, while the male posses another kind of habit, unknown as far we know.

#### Etymology.

The specific epithet is Latin and refers to the bifurcate setae of sternite 5 of the male.

## Supplementary Material

XML Treatment for Palpilongus

XML Treatment for Palpilongus bifurcus
